# The relationship between toxic heavy metal exposure and migraine and the modulatory role of an anti-inflammatory diet: A population-based cross-sectional study

**DOI:** 10.1097/MD.0000000000048475

**Published:** 2026-04-24

**Authors:** Zheng-Quan Zhang, Yong Zhang, Mi Zhang, Shuang-Shuang Zhu, Shuang-Jing Cai, Fang-Wang Fu, Lan-Bing Zhu, Pi-Guang Yao

**Affiliations:** a Department of Neurology, Wenzhou Dongtou People’s Hospital, Wenzhou, Zhejiang, China; b Department of Neurology, The Second Affiliated Hospital and Yuying Children’s Hospital of Wenzhou Medical University, Wenzhou, Zhejiang, China.

**Keywords:** cadmium, dietary, heavy metal, inflammation, lead, migraine

## Abstract

The influence of chronic, low-level environmental toxic metal exposure on migraine is poorly characterized. This study aimed to investigate the association between blood cadmium and lead levels and risk of migraine and how the inflammatory potential of dietary modified this association among United States adults. This cross-sectional study recruited 10,763 participants aged ≥ 20 from the National Health and Nutrition Examination Survey. The concentrations of blood cadmium and lead were measured using atomic absorption spectroscopy. The dietary inflammatory index (DII) assesses the inflammatory potential of diets and categorizes them into 3 groups: anti-inflammatory diets, low-intensity pro-inflammatory diets, and high-intensity pro-inflammatory diets. Migraine was diagnosed when participants reported that they had severe headaches or migraines during the past 3 months. Weighted multivariable logistic regression and restricted cubic spline models were used to determine the association of blood cadmium and lead levels, DII categories, and risk of migraine. The study included a total of 10,763 participants, of whom 2202 (20.5%) were diagnosed with migraine. After multivariable adjustment, blood cadmium levels were independently associated with an increased odds of migraine in a linear dose-response manner (odds ratio 1.18, 95% confidence interval 1.06–1.31, *P* = .004, *P* for nonlinearity = 0.064). Compared with participants having blood cadmium levels ≤ 0.3 μg/L, those with levels ≥ 0.7 μg/L had 21% higher odds of migraine (odds ratio 1.21, 95% confidence interval 1.01–1.46, *P* = .035). Mechanistic exploration analysis suggests that blood cadmium levels were associated with an increase in system inflammation response index and systemic immune-inflammation index, which reflect systemic inflammation, in migraineurs. Blood lead levels were not related with migraine, system inflammation response index, and systemic immune-inflammation index. Stratified analysis by the DII categories showed that the association between blood cadmium levels and odds of migraine remained significant only in the low-intensity pro-inflammatory diet subgroup, but disappeared in the anti-inflammatory diet and high-intensity pro-inflammatory diet subgroups. Higher blood cadmium levels are associated with an increased probability of suffering migraine, which could be mitigated by anti-inflammatory diets. Further studies are needed to clarify how cadmium triggers migraine attacks and to ascertain whether dietary interventions reduce risk of migraine in high-exposure populations.

## 1. Introduction

Migraine is a common chronic neurovascular disorder characterized by recurring, often highly disabling attacks of severe headache, accompanied by nausea, vomiting, and photosensitivity and phonosensitivity, lasting for 4 to 72 hours.^[1]^ According to the Global Burden of Disease Study 2019, migraine ranks as the second leading cause of years lived with disability on a global scale.^[2]^ The global burden of migraine is evident from 2 key metrics: a cumulative lifetime risk of 17.5%, and a point prevalence indicating that 7.0% of the population suffers from migraine attacks on a daily basis.^[3]^ Given the substantial socioeconomic burdens imposed by migraine, elucidating its pathophysiology has become imperative. Although the underlying mechanisms remain incompletely elucidated, neuroinflammation and oxidative stress have been established as critical contributors.^[4]^

Low-level and chronic environmental exposure to toxic metals, especially cadmium and lead, have recently received intense attention due to their widespread presence and adverse health effects.^[5]^ Humans are exposed to contaminant metals through air, water, soil, food, and extensive industrial and public use.^[6]^ Cadmium and lead can be efficiently absorbed through the respiratory and gastrointestinal tracts, however, the excretion of these metals has half-lives of decades.^[7]^ Global monitoring reveals sustained chronic exposure to cadmium and lead, with daily intake levels remaining consistently high at tens of micrograms per capita worldwide in recent years.^[5]^ The robust evidence has showed that cadmium and lead interfere with critical intracellular reactions and functions, leading to oxidative stress and chronic inflammation that result in increased risk of cardiovascular diseases, metabolic syndrome, and mortality.^[8,9]^ By contrast, the role of metal toxicity in migraine attacks is an emerging topic and has not been fully clarified. Given the adverse health effects of toxic metals, we hypothesized that low-level environmental exposure to cadmium and lead was positively associated with risk of migraine.

Dietary patterns, as a crucial component of daily lifestyle, are closely associated with health and disease due to the presence of nutrients, beneficial components, harmful substances, or contaminants in food.^[10]^ Fasting, alcohol, chocolate, cheese, and caffeine are frequently reported dietary triggers for migraine attacks.^[11]^ More health-conscious dietary patterns have also been associated with decreased migraine attack frequency and attenuated headache severity in clinical observations.^[12,13]^ Interventional evidences have demonstrated the efficacy of dietary approaches to stop hypertension and ketogenic diet in reducing migraine frequency and severity.^[14]^ On the other hand, essential metals, vitamins, phytochemicals, and probiotics in food have been shown to mitigate the adverse effects of toxic metals on human health through multiple mechanisms, including antagonizing cadmium and lead toxicity, reducing their absorption, and enhancing excretion.^[15]^ Thus, assuming the established association between cadmium or lead and migraine, dietary patterns may modulate this association. As inflammation may underlie the pathophysiology linking cadmium or lead toxicity to migraine, we characterized dietary patterns using the dietary inflammatory index (DII), a tool developed to assess the inflammatory potential of dietary based on the effects of foods and nutrients on inflammatory biomarkers (pro- or anti-inflammatory).^[16]^

In this study, we aimed to investigate the association between blood cadmium and lead levels and risk of migraine in a nationally representative sample of United States adults. Moreover, we analyzed how the DII modified this association. This study thereby addresses a critical knowledge gap regarding the complex relationships between nonoccupational toxic metal exposure, anti-inflammatory diets, and migraine.

## 2. Methods

### 2.1. Participants

This cross-sectional investigation utilized data from a nationally representative sample provided by the National Health and Nutrition Examination Survey (NHANES). Administered by the National Center for Health Statistics, NHANES is a biennial program that employs a complex, multistage probability design to recruit a representative cohort of noninstitutionalized US citizens. Participants underwent in-home interviews followed by standardized physical examinations at a mobile examination center (MEC) to evaluate the health and nutritional status of the population.

**Figure 1.**
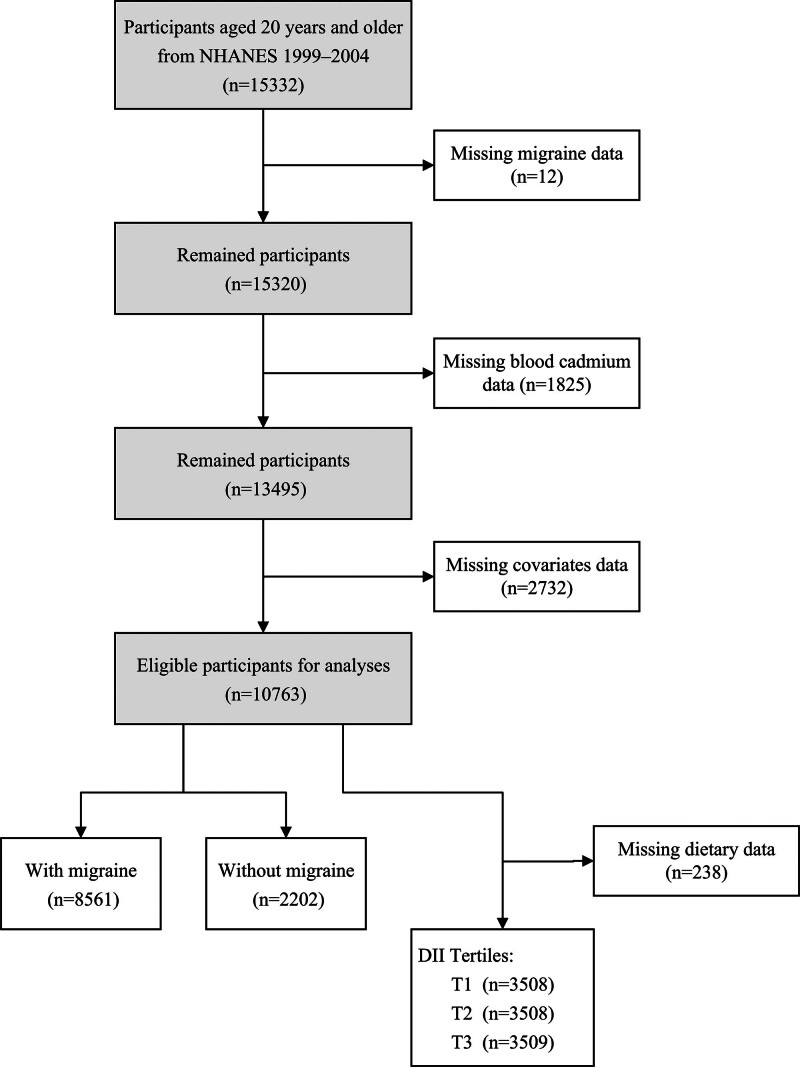
Study flow diagram. n = number of participants.

The NHANES 1999 to 2002 cycles provide data about headache questionnaires and blood cadmium and lead. Eligibility for analysis was confined to participants aged 20 years and above, who were the target population for the headache questionnaires. Those lacking complete data for migraine, blood cadmium, blood lead, or essential covariates were excluded, resulting in a final enrollment of 10,763 individuals (Fig. 1). The NHANES protocols were approved by the National Center for Health Statistics Ethics Review Board, and informed consent was secured from all subjects.

### 2.2. Blood cadmium and lead measurement

Blood cadmium and lead levels were measured using atomic absorption spectroscopy.^[17]^ Cadmium and lead quantification is based on the measurement of light absorbed at 228.8 nm and 283.3 nm, respectively, by ground-state atoms of cadmium and lead from either an electrodeless discharge lamp or by a hollow cathode lamp source. The cadmium and lead contents are determined on a PerkinElmer Model SIMAA 6000 simultaneous multielement atomic absorption spectrometer with Zeeman background correction.

### 2.3. Assessment of DII

Shivappa et al designed and developed the DII by examining the relationship between 45 different food parameters and inflammatory cytokines.^[16]^ Briefly, each food parameter was assigned an inflammatory effect score, with positive values indicating pro-inflammatory effects and negative values indicating anti-inflammatory effects. The intake of each food parameter for an individual was converted into a Z-score by subtracting the global daily mean intake and dividing by its standard deviation. This Z-score was then transformed into a percentile score, multiplied by 2, and then subtracted by 1. Subsequently, the centered percentile for each food parameter was multiplied by its corresponding “food parameter-specific inflammatory effect score” to obtain the “food parameter-specific DII score.” Finally, all food parameter-specific DII scores were summed to derive the individual’s “overall DII score.” A positive score indicates pro-inflammatory potential of the diet, a negative score represents anti-inflammatory potential, and a zero score denotes no significant effect.

Since NHANES did not report all food parameters, we included 26 food parameters: alcohol, vitamin B_12_, vitamin B_6_, β-carotene, caffeine, carbohydrate, cholesterol, energy, total fat, dietary fiber, folic acid, iron, magnesium, monounsaturated fatty acid, niacin, protein, polyunsaturated fatty acid, vitamin B_2_, saturated fat, selenium, vitamin B_1_, vitamin A, vitamin C, vitamin D, vitamin E, and zinc. Shivappa et al reported that using no more than 30 food parameters remains sufficient to preserve the DII’s predictive validity for diet-related inflammation.^[18]^ The intake of each food parameter was derived from a single 24-hour dietary recall interview, which recorded the types and quantities of food consumed in the 24 hours prior to the interview.^[19]^

### 2.4. Diagnosis of migraine

We determined migraine status based on responses to the Miscellaneous Pain section of the survey, which was utilized in the home interview to assess various pain conditions. The question: “During the past 3 months, did you have severe headaches or migraines?” was included in this questionnaire. The American Migraine Prevalence and Prevention study demonstrated that approximately 94% of individuals with self-reported severe headaches are diagnosed with migraine or probable migraine according to the second edition of the International Classification of Headache Disorders- criteria.^[20]^ We classified participants responding “yes” as having migraine; conversely, those answering “no” were designated as the control group.

### 2.5. Covariates

During the home interview, participants were asked to answer the demographics questionnaire (age, sex, race, education level, marital status, and family income-to-poverty ratio) and questions regarding the presence of different medical conditions (hypertension, diabetes mellitus, and hyperlipidemia). On average, 2 weeks after home interview, participants were scheduled for examinations in the MEC. Physical measurements (body mass index [BMI] and blood pressure) were recorded by a trained examiner. Blood samples were collected to assess fasting glucose, glycohemoglobin, total cholesterol, and low-density lipoprotein cholesterol levels and blood cell count. Alcohol and cigarette use was inquired during the MEC interview.

The definitions for hypertension, diabetes mellitus, hyperlipidemia, along with the definitions for smoking history and alcohol use, were aligned with those used in prior research.^[21]^ The system inflammation response index (SIRI) is calculated using the following formula: SIRI = (Neutrophils (10^9^/L) × Monocytes (10^9^/L))/ Lymphocytes (10^9^/L). The systemic immune-inflammation index (SII) is calculated using the following formula: SII = (Platelets (10^9^/L) × Neutrophils (10^9^/L))/ Lymphocytes (10^9^/L).

### 2.6. Statistical analysis

Sample weights were considered to illustrate the complex sampling design of NHANES. According to the NHANES analytic guidelines, the sample weights for 1999 to 2002 were calculated as 2/3 × WTMEC4YR, while those for 2003 to 2004 were computed as 1/3 × WTMEC2YR.^[22]^ Continuous variables were summarized as weighted medians with interquartile ranges and differences between migraine and control groups were evaluated using the Wilcoxon rank-sum test. Categorical variables were summarized using the unweighted number of cases and the weighted percentage. Group comparisons for these variables were performed using the chi-square test, incorporating Rao and Scott second-order correction. For normally distributed continuous variables, associations were assessed via Pearson correlation analysis. Due to the non-normal distribution of blood cadmium and lead levels, we applied natural logarithmic transformation to these variables.

The association of blood cadmium and lead with migraine was evaluated by logistic regression analyses. Blood cadmium or lead was entered into the models as the quartile-based categorical variable and odds ratio (OR) was compared across quartiles, with the first quartile serving as the reference group. The multivariable Model 1 was adjusted for age, sex, and race, and the multivariable Model 2 was adjusted for age, sex, race, education level, marital status, family income-to-poverty ratio, smoking, drinking, BMI, hypertension, diabetes mellitus, and hyperlipidemia. Subgroup analyses stratified by sex (female/male) were performed because a 2-fold higher prevalence of migraine was observed in females compared to males.^[23]^ Furthermore, to visualize the potential nonlinear associations, we fitted restricted cubic spline models for blood cadmium and lead levels in relation to migraine. The splines were specified with 4 knots (at the 5th, 35th, 65th, and 95th percentiles), using the median of the lowest quartile as the reference point.

To determine whether blood cadmium or lead is associated with inflammation in migraine, we performed linear regression analyses to examine the associations of blood cadmium and lead with SIRI and SII, which are excellent markers of systemic inflammation.^[24]^ Since the residual distributions of the SIRI and SII models did not meet the assumption of normality, these indices were natural log-transformed prior to model construction. The models were adjusted for the same covariates in Model 2. To investigate the moderating effect of DII on the association between blood cadmium and migraine, we performed subgroup analyses across the different levels of DII score. Participants with a DII score < 0 were classified as the anti-inflammatory diet group, while those with a DII score > 0 were divided into 2 subgroups based on the median value, representing the low-intensity pro-inflammatory diet group and the high-intensity pro-inflammatory diet group, respectively. Data analysis was conducted in R version 4.2.1 (R Foundation for Statistical Computing, Vienna, Austria). Statistical significance was defined as a 2-sided *P* value < .05.

## 3. Results

### 3.1. Participants characteristics

Table 1 summarizes the baseline characteristics of the study population. Of the 10,763 participants, 20.5% (n = 2202) reported migraine within the preceding 3 months. The overall cohort had a median age of 44.0 years (IQR: 33.0–57.0) and was 51.5% female. Comparative analysis revealed that participants with migraine were significantly younger, had a higher proportion of females and nonwhite individuals, and exhibited lower educational attainment and economic status compared to the non-migraine group. Additionally, the migraine group had higher BMI values but lower rates of alcohol consumption and diabetes mellitus (all *P* < .05).

**Table 1:** Characteristics of participants with and without migraine.

Characteristic	Overall (N = 10763)	Without migraine (N = 8561)	With migraine (N = 2202)	*P* value
Age, years	44.0 (33.0–57.0)	46.0 (34.0–60.0)	41.0 (31.0–51.0)	< .001
Sex, %				< .001
Female	5554 (51.5)	4091 (47.6)	1463 (65.3)	
Male	5209 (48.5)	4470 (52.4)	739 (34.7)	
Race, %				.010
Hispanic	2843 (12.3)	2209 (11.8)	634 (14.1)	
Non-Hispanic White	5645 (73.7)	4596 (74.7)	1049 (70.0)	
Non-Hispanic Black	1916 (9.7)	1476 (9.3)	440 (11.3)	
Other Race	359 (4.3)	280 (4.2)	79 (4.6)	
Education level, %				< .001
Less than high school	3301 (19.0)	2559 (17.9)	742 (22.8)	
High school	2539 (26.0)	1991 (25.5)	548 (27.6)	
More than high school	4923 (55.0)	4011 (56.6)	912 (49.6)	
Marital status, %				.57
Married/living with partner	6835 (65.8)	5481 (66.1)	1354 (64.7)	
Widowed/divorced/separated	2320 (18.3)	1859 (18.0)	461 (19.0)	
Never married	1608 (15.9)	1221 (15.8)	387 (16.3)	
PIR, %				< .001
< 1.3	2988 (20.6)	2201 (18.4)	787 (28.5)	
1.3–3.5	4170 (36.0)	3326 (35.6)	844 (37.6)	
> 3.5	3605 (43.4)	3034 (46.1)	571 (33.9)	
Smoking, %	5278 (50.1)	4225 (50.0)	1053 (50.7)	.63
Drinking, %	6140 (63.1)	4984 (64.7)	1156 (57.6)	< .001
BMI, kg/m2	27.1 (23.8–31.3)	27.0 (23.8–31.0)	27.7 (23.9–32.6)	< .001
Hypertension, %	4097 (32.9)	3312 (33.0)	785 (32.3)	.51
DM, %	1400 (9.1)	1144 (9.4)	256 (8.1)	.022
Hyperlipidemia, %	6666 (60.3)	5377 (60.8)	1289 (58.4)	.15
SIRI	1.1 (0.8–1.6)	1.1 (0.8–1.6)	1.1 (0.7–1.6)	.38
SII	530.0 (385.0–733.3)	523.9 (382.4–728.0)	550.0 (394.1–751.1)	.006
Cadmium, μg/L	0.4 (0.2–0.7)	0.4 (0.2–0.6)	0.4 (0.2–0.7)	.017
Lead, μg/dL	1.6 (1.0–2.4)	1.6 (1.1–2.4)	1.4 (0.9–2.2)	< .001
DII*	1.4 (-0.2–2.6)	1.2 (-0.3–2.5)	1.8 (0.2–2.9)	< .001

BMI = body mass index, DII = dietary inflammatory index, DM = diabetes mellitus, L = litre, PIR = family income-to-poverty ratio, SII = systemic immune-inflammation index, SIRI = system inflammation response index, dL = decilitre, kg = kilogram, μg = microgram.

* missing 238 cases.

### 3.2. Association of blood cadmium and lead with migraine

**Figure 2.**
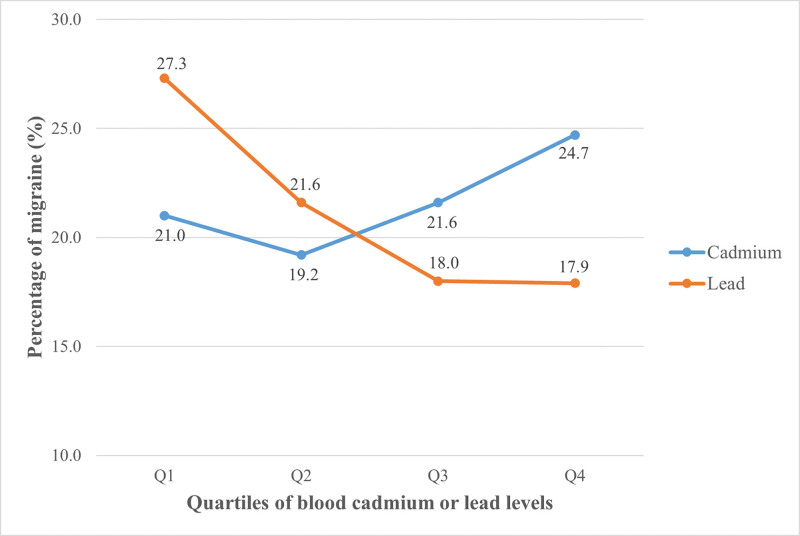
Prevalence of migraine according to the quartiles of blood cadmium and lead levels.

The average blood cadmium and lead levels across all participants were 0.4 (0.2–0.7) μg/L and 1.6 (1.0–2.4) μg/dL, respectively. There was a weak correlation between log-transformed blood cadmium and lead levels (*R* = 0.34, *P* < .001). The blood cadmium levels in migraineurs were higher than those in non-migraineurs (*P* = .017), whereas the blood lead levels showed the opposite trend (*P* < .001) (Table 1). The prevalence of migraine was lowest (19.2%) in the second quartile of blood cadmium levels and increased to 24.7% in the highest quartile. By contrast, the prevalence of migraine decreased gradually from 27.3% in the lowest quartile of blood lead levels to 17.9% in the highest quartile (Fig. 2).

**Figure 3.**
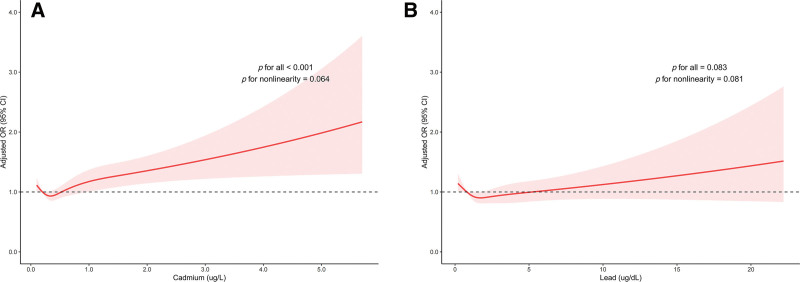
Restricted cubic spline plots of blood (A) cadmium and (B) lead levels in relation to risk of migraine. Adjusted for the same covariates in Model 2. CI = confidence interval, L = litre, OR = odds ratio, dL = decilitre, μg = microgram.

After fully adjusting for potential confounders, blood cadmium levels were significantly associated with higher odds of migraine (OR 1.18, 95% confidence interval [CI] 1.06–1.31, *P* = .004), whereas no significant association was observed between blood lead levels and migraine (OR 1.02, 95% CI 0.98–1.05, *P* = .29). Compared to participants in the lowest quartile, those in the highest quartile of blood cadmium levels (≥ 0.7 μg/L) had 21% higher odds of migraine (OR 1.21, 95% CI 1.01–1.46, *P* = .035) (Table 2). The restricted cubic spline analyses further confirmed a linear positive association between blood cadmium levels and risk of migraine (*P* for all < .001 and *P* for nonlinearity = .064), while no significant association was observed for blood lead levels (*P* for all = .083 and *P* for nonlinearity = .081) (Fig. 3). In the sex-stratified analysis, blood cadmium levels were significantly associated with higher odds of migraine both in females and males (OR 1.17, 95% CI 1.01–1.36, *P *= .037 for females; OR 1.18, 95% CI 1.02–1.36, *P* = .023 for males; Figure S1, Supplemental Digital Content, https://links.lww.com/MD/R772).

**Table 2:** Logistic regression analyses to identify the association between blood cadmium and lead levels and risk of migraine.

	Migraine/Total	Adjusted Model 1		Adjusted Model 2	
		OR (95% CI)	*P* value	OR (95% CI)	*P* value
Cadmium, μg/L		1.26 (1.15–1.38)	< .001	1.18 (1.06–1.31)	.004
Quartile of cadmium					
Quartile 1 (≤ 0.3 μg/L)	832/4067 (21.0%)	Ref		Ref	
Quartile 2 (0.4 μg/L)	362/1841 (19.2%)	0.94 (0.75–1.18)	.59	0.93 (0.74–1.18)	.54
Quartile 3 (0.5–0.6 μg/L)	410/2167 (21.6%)	1.18 (0.97–1.43)	.10	1.10 (0.90–1.36)	.34
Quartile 4 (≥ 0.7 μg/L)	598/2688 (24.7%)	1.38 (1.17–1.61)	< .001	1.21 (1.01–1.46)	.041
* P* for trend		< .001		.018	
Lead, μg/dL		1.03 (1.00–1.06)	.066	1.02 (0.98–1.05)	.29
Quartile of lead					
Quartile 1 (≤ 1.1 μg/dL)	785/2916 (27.3%)	Ref		Ref	
Quartile 2 (1.2–1.7 μg/dL)	568/2645 (21.6%)	0.99 (0.85–1.15)	.89	0.98 (0.83–1.15)	.79
Quartile 3 (1.8–2.7 μg/dL)	447/2680 (18.0%)	0.97 (0.78–1.21)	.81	0.92 (0.73–1.16)	.47
Quartile 4 (≥ 2.8 μg/dL)	402/2522 (17.9%)	1.11 (0.87–1.41)	.40	1.00 (0.80–1.26)	.97
* P* for trend		.47		.90	

Model 1: adjusted for age, sex, and race.

Model 2: adjusted for age, sex, race, education level, marital status, PIR, smoking, drinking, BMI, hypertension, diabetes mellitus, and hyperlipidemia.

BMI = body mass index, CI = confidence interval, L = litre, OR = odds ratio, PIR = family income-to-poverty ratio, dL = decilitre, μg = microgram.

### 3.3. Association of blood cadmium and lead with systemic inflammation

The SII was higher in migraineurs compared to non-migraineurs (550.0 [394.1–751.1] vs 523.9 [382.4–728.0], *P* = .006), while the SIRI was comparable between the 2 groups (1.1 [0.7–1.6] vs 1.1 [0.8–1.6], *P* = .38) (Table 1). Based on the hypothesis that inflammation plays a role in the pathogenesis of migraine,^[4]^ we investigated the association of blood cadmium and lead with systemic inflammation in migraineurs. When analyzed as a continuous variable, each 1 μg/L increase in blood cadmium was associated with an average increase of 0.05 in log-transformed SIRI (β 0.05, 95% CI 0.01–0.08, *P* = .007). When analyzed as a categorical variable, the highest quartile of blood cadmium showed nonsignificant increases in log-transformed SIRI (β 0.07, 95% CI −0.01–0.14, *P* = .092) and SII (β 0.06, 95% CI 0.00–0.12, *P* = .056) compared to the lowest quartile. Regardless of whether analyzed as a continuous or categorical variable, blood lead showed no association with log-transformed SIRI or SII (Table 3).

**Table 3:** Linear regression analyses to identify the associations of blood cadmium and lead levels with log-transformed SIRI and SII.

	Ln(SIRI)		Ln(SII)	
	Beta (95% CI)	*P* value	Beta (95% CI)	*P* value
Cadmium, μg/L	0.05 (0.01–0.08)	.007	0.02 (-0.01–0.06)	.19
Quartiles of cadmium				
Quartile 1 (≤ 0.3 μg/L)	Ref		Ref	
Quartile 2 (0.4 μg/L)	−0.06 (−0.14–0.02)	.14	−0.01 (−0.09–0.07)	.84
Quartile 3 (0.5–0.6 μg/L)	0.02 (−0.07–0.12)	.62	0.03 (−0.06–0.11)	.53
Quartile 4 (≥ 0.7 μg/L)	0.07 (−0.01–0.14)	.092	0.06 (0.00–0.12)	.056
* P* for trend	.033		.053	
Lead, μg/dL	0.00 (−0.01–0.02)	.54	0.00 (−0.01–0.01)	.72
Quartiles of lead				
Quartile 1 (≤ 1.1 μg/dL)	Ref		Ref	
Quartile 2 (1.2–1.7 μg/dL)	−0.04 (−0.14–0.05)	.38	−0.03 (−0.11–0.05)	.44
Quartile 3 (1.8–2.7 μg/dL)	0.04 (−0.06–0.15)	.42	0.02 (−0.06–0.10)	.57
Quartile 4 (≥ 2.8 μg/dL)	0.00 (−0.11–0.11)	.98	−0.05 (−0.13–0.04)	.27
* P* for trend	.61		.51	

Adjusted for age, sex, race, education level, marital status, PIR, smoking, drinking, BMI, hypertension, diabetes mellitus, and hyperlipidemia.

BMI = body mass index, CI = confidence interval, L = litre, PIR = family income-to-poverty ratio, SII = systemic immune-inflammation index, SIRI = system inflammation response index, dL = decilitre, μg = microgram.

### 3.4. DII modifies the association between blood cadmium and migraine

After excluding participants with incomplete dietary data, 10,525 individuals were retained for DII-related analyses. The average DII score across all participants was 1.4 (−0.2–2.6).Among 10,525 participants, 2775 (26.4%) had anti-inflammatorydiets (DII < 0) and 7750 (73.6%) had pro-inflammatory diets. Migraineurs had significantly higher DII scores compared to non-migraineurs (1.8 [0.2–2.9] vs 1.2 [-0.3–2.5], *P* < .001) (Table 1). Weak but statistically significant correlations were observed between DII scores and log-transformed blood cadmium (*R* = 0.14, *P* < .001) and lead levels (r = −0.03, *P* < .001). Participants were stratified by DII scores with the following cutoff values: anti-inflammatory: DII < 0, low-intensity pro-inflammatory: 0 ≤ DII ≤ 2.09, and high-intensity pro-inflammatory: DII ≥ 2.10. These participants with high DII scores exhibited significantly higher SII levels (Figure S2, Supplemental Digital Content, https://links.lww.com/MD/R772).

**Figure 4.**
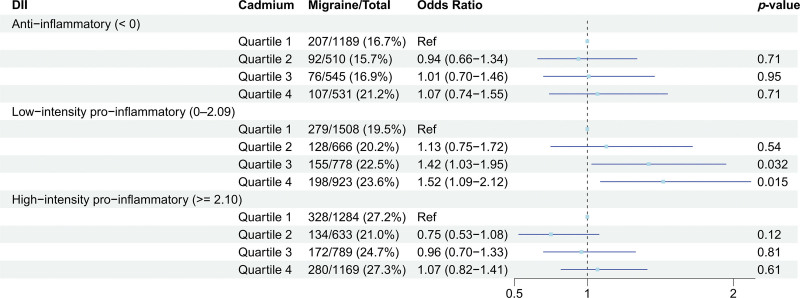
Subgroup analyses to identify the moderating effects of inflammatory potential of diets on the association between blood cadmium levels and risk of migraine. Adjusted for the same covariates in Model 2.

Based on the associations of blood cadmium with migraine and systemic inflammation, we further explored the association between blood cadmium levels and risk of migraine across different DII scores. When further adjusting for DII score in Model 2, both blood cadmium levels (OR 1.08, 95% CI 1.06–1.31, *P* = .004) and DII score (OR 1.05, 95% CI 1.02–1.08, *P* = .005) were positively associated with odds of migraine (Table S1, Supplemental Digital Content, https://links.lww.com/MD/R771). In the stratified analysis by the DII categories, the association between blood cadmium levels and odds of migraine remained significant only in the low-intensity pro-inflammatory diet subgroup (OR 1.42, 95% CI 1.03–1.95, *P *= .032 for quartile 3; OR 1.52, 95% CI 1.09–2.12, *P* = .015 for quartile 4; Fig. 4). In the anti-inflammatory diet and high-intensity pro-inflammatory diet subgroups, no significant association was observed between blood cadmium and migraine.

## 4. Discussion

Using a nationally representative cross-sectional survey, we identify that higher cadmium exposure was significantly associated with increased odds of migraine after adjustment for confounding factors in United States adults. An increase in blood cadmium levels is associated with elevated systemic inflammation levels in migraineurs. Blood lead was not related with migraine and systemic inflammation. A dietary pattern with anti-inflammatory potential mitigated the increased risk of migraine associated with elevated blood cadmium levels.

In a previous small-sample study, the blood cadmium and lead levels in 25 migraineurs during an attack were higher than those in 25 healthy controls.^[25]^ Another small-sample study found that the blood lead levels in 50 migraineurs during an attack were higher than those in 50 healthy controls, while the blood cadmium levels were similar between the 2 groups.^[26]^ However, these studies have limitations, including small-sample sizes that lack representativeness, imbalanced sex ratios (12% male and 26% male, respectively), and a failure to account for confounding biases. In contrast, univariate analysis showed that blood lead levels were higher in the control group in our study, potentially owing to the group’s older mean age and greater male predominance. After adjusting for the aforementioned confounding factors, blood lead was not related with migraine. A major strength of this study is the use of a large, nationally representative sample to link chronic low-level environmental metal exposure with migraine. However, the mechanisms underlying the association between cadmium exposure and migraine are still unknown.

Migraine is understood as a neurovascular disorder characterized by the activation and sensitization of the trigeminovascular system, a process that can be initiated by cortical spreading depolarization.^[27]^ Activation of the trigeminovascular system prompts the release of various neuropeptides and neurotransmitters (e.g., calcitonin gene-related peptide, substance P, vasoactive intestinal peptide, nitric oxide, histamine) from neurons, glial cells, and mast cells, culminating in neurogenic inflammation. This inflammatory state is primarily characterized by enhanced vascular permeability, leukocyte infiltration, activation of glial cells, and an upregulation of inflammatory mediators including cytokines and oxygen radicals.^[4]^ Compared with healthy controls, migraineurs exhibit elevated serum levels of pro-inflammatory cytokines, including interleukin (IL)-1β, IL-6, and tumor necrosis factor-α. Moreover, these cytokine levels are further increased during the ictal phase of migraine attacks.^[28]^ Thus, neurogenic inflammation and oxidative stress are major contributors to migraine attacks.

It is generally accepted that the key mechanisms underpinning heavy metals toxicity are oxidative stress and inflammation induction. On one hand, cadmium displaces iron from various cytosolic and membrane-bound proteins, thereby increasing the pool of freely available Iron ions. These ions participate in Fenton reactions to generate reactive oxygen species while simultaneously promoting reactive nitrogen species production.^[29]^ On the other hand, cadmium binds to sulfhydryl groups and potently inhibits the activity of key antioxidant enzymes, including superoxide dismutase, catalase, and glutathione peroxidase, ultimately leading to depletion of the cellular antioxidant defense system.^[9]^ In addition, cadmium exposure in vitro, in rodents, and in humans correlate with increased release of pro-inflammatory cytokines and inflammatory mediators, such as IL-1β, IL-6, tumor necrosis factor-α, matrix metalloproteinases, and cyclooxygenase-2.^[30]^ Our study utilized systemic inflammatory response indices (SIRI and SII) as biomarkers, and the results provide robust evidence supporting the pro-inflammatory role of cadmium exposure in humans. These pro-inflammatory and pro-oxidative roles may explain the positive correlation between blood cadmium levels and risk of migraine.

In response to the adverse effects of cadmium on the migraine attacks, we need to implement relevant public health measures through legislation and strengthen health education for the public, thereby minimizing toxic metals exposure. Despite these efforts, low-level toxic metals exposure likely remains pervasive and additional interventions are necessary to alleviate heavy metal toxicity. Our findings suggest that an anti-inflammatory dietary pattern is associated with lower systemic inflammation levels and mitigates the increased risk of migraine associated with cadmium exposure. Healthier dietary patterns typically provide higher intake of antioxidants, including essential trace elements and vitamins, which alleviate cadmium toxicity.^[15]^ Notably, the adverse effects of heavy metals on cognitive function in elderly individuals were observed only among those with pro-inflammatory diets but disappeared in anti-inflammatory diet adherents, supporting the protective role of anti-inflammatory diets against metal toxicity.^[31]^ Interestingly, in our study, cadmium exposure showed no association with migraine in the high-intensity pro-inflammatory diet subgroup, possibly because the strong pro-inflammatory effects of dietary dominated over cadmium contribution to migraine pathogenesis. Other intervention measures should also be emphasized, such as chelation therapy and physical activity. Chelation therapy using ethylenediaminetetraacetic acid is an effective strategy to remove toxic metals, especially cadmium, from the human body.^[32]^ The metal toxicity and adverse health effects of cadmium can also be mitigated by enhancing physical activity.^[33,34]^

We acknowledge several limitations that should be considered. Firstly, migraine status was derived from participant self-report as opposed to a clinical evaluation based on International Classification of Headache Disorders criteria. While this method for identifying migraine has been employed in previous research,^[21,35,36]^ it may have inadvertently included a subset of individuals with non-migraine headaches who did not strictly meet the formal diagnostic criteria. Second, this study did not distinguish between migraine patients during attack and interictal periods. Assuming that acute exposure or release from accumulated tissue deposits may trigger migraine attacks, whether patients during migraine attacks exhibit higher blood cadmium levels than those in interictal periods warrants further investigation. Third, the cross-sectional nature of this study precludes the determination of causal relationships. Nevertheless, considering the considerably long biological half-lives of cadmium and lead, blood cadmium and lead levels can serve as reliable biomarkers reflecting long-term cumulative exposure. Future research should employ prospective cohort designs to clarify the potential causal links between exposure to toxic metals, adherence to anti-inflammatory diets, and the incidence of migraine attacks.

## 5. Conclusions

The investigation utilized a nationally representative sample and indicates that low-level and chronic environmental exposure of cadmium is associated with elevated systemic inflammation and risk of migraine. Adherence to an anti-inflammatory diet contributes to mitigating the increased risk of migraine associated with cadmium exposure. Further studies are warranted to elucidate the precise mechanisms by which cadmium exposure triggers migraine attacks and to validate the efficacy of dietary interventions in alleviating migraine episodes among high-risk populations with cadmium exposure in prospective cohorts.

## Author contributions

**Conceptualization:** Pi-Guang Yao.

**Formal analysis:** Zheng-Quan Zhang, Shuang-Shuang Zhu.

**Data curation:** Yong Zhang.

**Investigation:** Yong Zhang, Mi Zhang, Shuang-Jing Cai.

**Methodology:** Zheng-Quan Zhang, Pi-Guang Yao.

**Project administration:** Pi-Guang Yao.

**Resources:** Yong Zhang, Shuang-Jing Cai.

**Validation:** Mi Zhang.

**Visualization:** Zheng-Quan Zhang.

**Writing – original draft:** Zheng-Quan Zhang.

**Writing – review & editing:** Yong Zhang, Mi Zhang, Shuang-Shuang Zhu, Shuang-Jing Cai, Fang-Wang Fu, Lan-Bing Zhu, Pi-Guang Yao.

## Supplementary Material




