# Supplementary material for: The relationship between toxic heavy metal exposure and migraine and the modulatory role of an anti-inflammatory diet: A population-based cross-sectional study
Source: Medicine (Baltimore). 2026 Apr 24;105(17):e48475. doi: 10.1097/MD.0000000000048475 (PMC13124364; doi:10.1097/MD.0000000000048475)
Supplement: Supplementary file 1 [file medi-105-e48475-s001.pdf]

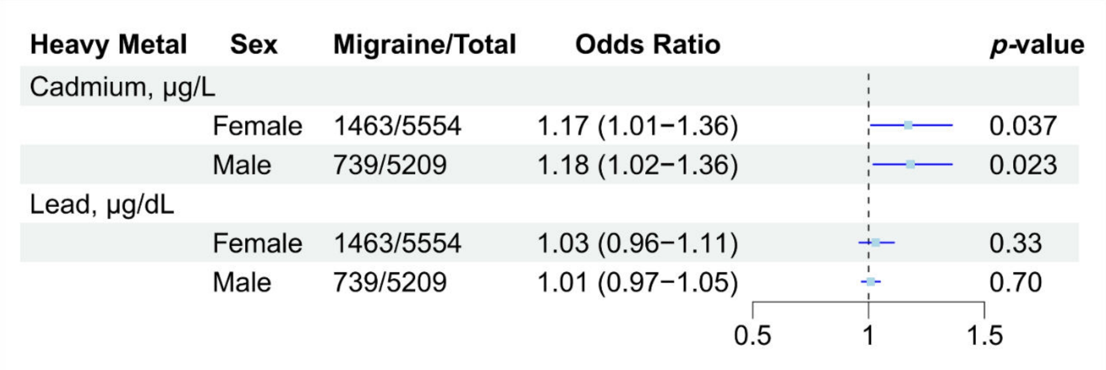

Heavy Metal	Sex	Migraine/Total	Odds Ratio	*p*-value
Cadmium, µg/L				
	Female	1463/5554	1.17 (1.01−1.36)	0.037
	Male	739/5209	1.18 (1.02−1.36)	0.023
Lead, µg/dL				
	Female	1463/5554	1.03 (0.96−1.11)	0.33
	Male	739/5209	1.01 (0.97−1.05)	0.70

Figure S1. Subgroup analyses to identify the association between blood cadmium and lead levels and risk of migraine in females and males.

Adjusted for the same covariates in Model 2.

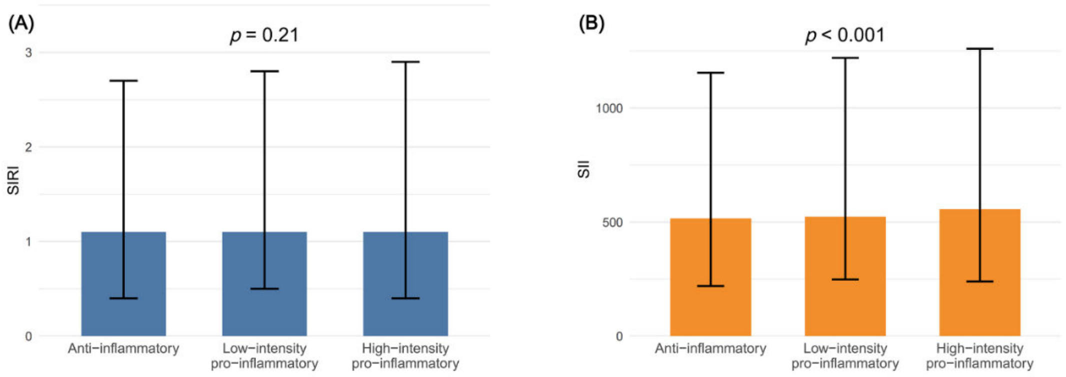

Figure S2. Comparison of the SIRI and SII levels in different inflammatory potential of diets.
