# Supplementary material for: The relationship between toxic heavy metal exposure and migraine and the modulatory role of an anti-inflammatory diet: A population-based cross-sectional study
Source: Medicine (Baltimore). 2026 Apr 24;105(17):e48475. doi: 10.1097/MD.0000000000048475 (PMC13124364; doi:10.1097/MD.0000000000048475)
Supplement: Supplementary file 2 [file medi-105-e48475-s002.pdf]

Table S1. Logistic regression analyses to identify the association between blood cadmium levels, DII scores, and risk of migraine

**Continuous variables**	**OR (95% CI)**	***p*-value**	**Categorical variables**	**OR (95% CI)**	***p*-value**
**Cadmium, μg/L**	1.18 (1.06–1.31)	0.003	**Quartiles of cadmium**		
			Quartile 1 (≤ 0.3 μg/L)	Ref	
			Quartile 2 (0.4 μg/L)	0.93 (0.74–1.19)	0.56
			Quartile 3 (0.5–0.6 μg/L)	1.12 (0.91–1.38)	0.26
			Quartile 4 (≥ 0.7 μg/L)	1.21 (1.01–1.46)	0.039
**DII**	1.05 (1.02–1.08)	0.005	**DII**		
			Anti-inflammatory (< 0)	Ref	
			Low-intensity pro-inflammatory (0–2.09)	1.10 (0.94–1.30)	0.22
			High-intensity pro-inflammatory (≥ 2.10)	1.19 (1.03–1.38)	0.020

Adjusted for age, sex, race, education level, marital status, PIR, smoking, drinking, BMI, hypertension, diabetes mellitus, and hyperlipidemia.

BMI, body mass index; CI, confidence interval; DII, dietary inflammatory index; OR, odds ratio; PIR, family income-to-poverty ratio.
